# A Comparison of Operative Time and Intraoperative Blood Volume Loss Between Stemless and Short‐stem Anatomic Total Shoulder Arthroplasty: A Single Institution's Experience

**DOI:** 10.5435/JAAOSGlobal-D-22-00141

**Published:** 2022-07-20

**Authors:** Konrad I. Gruson, Yungtai Lo, Savino Stallone, Feras Qawasmi, Sung Lee, Priyam Shah

**Affiliations:** From the Department of Orthopaedic Surgery, Montefiore Medical Center, Albert Einstein College of Medicine, Bronx, NY (Dr. Gruson, Stallone, Dr. Qawasmi, and Dr. Lee), and the Albert Einstein College of Medicine, Bronx, NY (Dr. Lo, Shah).

## Abstract

There has been increasing interest in the use of stemless humeral implants for total shoulder arthroplasty when compared with both short-stem (SS) and standard-length implants. Although evidence for decreased surgical time and blood loss exists for stemless versus standard-length stems, far less literature exists comparing these clinical parameters for stemless versus SS implants.

**Methods:**

A retrospective review of consecutive anatomic total shoulder arthroplasty (aTSA) cases conducted by a single, fellowship-trained shoulder surgeon was undertaken from January 2016 through January 2022 with the exception of March 2020 through January 2021 secondary to the COVID-19 pandemic. Demographic patient and surgical data, including age, sex, body mass index, American Society of Anesthesiologists score, age-adjusted Charlson Comorbidity Index, prior ipsilateral shoulder arthroscopy, surgical time, use of a Hemovac drain and/or tranexamic acid, hospital length of stay (LOS), and both postoperative day #1 (POD 1) and discharge visual analog scores. The use of a stemless or SS implant was recorded. Intraoperative total blood volume loss (TBVL) was calculated, in addition to the need for either intraoperative or postoperative transfusions. Nonparametric analysis of covariance was used to examine effects of stemless versus SS aTSA on surgical time and intraoperative TBVL adjusted for demographic, clinical, and surgical variables.

**Results:**

There were 47 SS and 83 stemless anatomic implants included, of which 74 patients (57%) overall were women. The median surgical time for the stemless cohort was 111 minutes (IQR 96-130) versus 137 minutes (IQR 113-169) for the SS cohort (*P* < 0.00001). The median intraoperative TBVL for the stemless cohort was 298.3 mL (IQR 212.6-402.8) versus 359.7 mL (IQR 253.9-415.0) for the SS cohort (*P* = 0.05). After multivariable regression analysis, use of stemless humeral implants was independently associated with both decreased surgical time and intraoperative blood loss (*P* < 0.001 and *P* = 0.005, respectively). There was a shorter median hospital LOS in the stemless group (2 days [IQR 1-2] versus 2 days [IQR 2-3], *P* = 0.03). The visual analog score pain score at discharge was lower among the stemless cohort (0 [IQR 0-3] versus 4 [IQR 2-6], *P* < 0.00001). Increased surgical time was associated with intraoperative TBVL (r = 0.340, *P* < 0.0001).

**Discussion:**

Stemless aTSA is associated with a markedly decreased surgical time and intraoperative TBVL when compared with a SS aTSA. Furthermore, the use of a stemless implant results in a shorter hospital LOS and lower discharge pain scores.

The use of stemless humeral implants in anatomic total shoulder arthroplasty has experienced increased interest relative to both standard-length (SL) and short-stem (SS) implants despite the dearth of evidence demonstrating its clinical superiority over stemmed arthroplasty.^1^ The theoretical benefits of a stemless device include ease of extraction during revision, preservation of the bone, faster surgical time, decreased blood loss, and concomitant reduced need for transfusion.^2^ Several previous studies have reported on decreased surgical time with stemless implants compared with SL anatomic stems,^3-5^ although the study by Malcherczyk et al^3^ was likely underpowered given the small patient numbers in the stemless cohort. Furthermore, several studies included both cemented and noncemented stems, which may have confounded their findings.^3,4^ More recently, Anastasio et al^6^ reported on a nearly 14-minute shorter surgical time for stemless compared with SS total shoulder arthroplasty, even when controlling for body mass index (BMI). By contrast, Wiater et al,^7^ in a multicenter randomized controlled trial comparing a stemless anatomic total shoulder arthroplasty (aTSA) with the same manufacturer's noncemented SS implant, found no difference in surgical time. Apart from BMI, male sex has also been demonstrated to be independently associated with increased surgical time during shoulder arthroplasty.^8^ However, the authors were unable to differentiate between primary reverse total shoulder arthroplasty (rTSA) and aTSA, in addition to the humeral stem length used, given the use of a database.^8^ The importance of minimizing surgical time during aTSA has been previously shown to reduce the risk for perioperative complications.^9,10^

The prevalence of blood transfusion after shoulder arthroplasty, as well as its associated risk factors, has previously been described.^11,12^ Given the varied institutional triggers for administering a transfusion, however, has resulted in a wide range of prevalence estimates. Several previous studies have reported a zero% prevalence of transfusion for patients undergoing stemless aTSA.^3,4^ A proposed advantage of stemless implants compared with SL implants has been a decrease in intraoperative blood loss given the avoidance of the need for humeral canal preparation. Berth et al^4^ found a markedly lower amount for blood loss in a cohort of stemless aTSA when compared with SL humeral stems. A recent meta-analysis demonstrated a nearly 100-mL lower blood loss among stemless implants compared with stemmed implants, although the included studies involved SL stems.^13^ The risk factors for blood loss in the setting of shoulder arthroplasty, including both rTSA and aTSA, have been demonstrated to be male sex, BMI, and surgical time.^3^ The relationship between total blood loss and need for transfusion has not been clearly demonstrated in comparative studies of stemless and SL aTSAs.^3^ Furthermore, there remains a dearth of literature examining the effect of using stemless humeral implants for aTSA on blood loss and need for transfusion when compared with the use of SS implants.

The primary purpose of this study was to determine whether there was a difference in surgical time and intraoperative total blood volume loss (TBVL) between stemless aTSA and SS implants. Secondary purposes of this study were to examine the relationship between humeral implant design and hospital length of stay (LOS) and both postoperative day 1 and discharge visual analog score (VAS) pain scores. We hypothesized that stemless implants would be associated with a markedly reduced surgical time, decreased intraoperative blood loss, and reduced postoperative pain.

## Methods

The demographic and surgical data for all consecutive primary anatomic total shoulder arthroplasties conducted by the senior author (KIG) at our institution were retrospectively reviewed and collected. All procedures were performed by a single fellowship-trained shoulder surgeon and involved an underlying diagnosis of osteoarthritis. At the time of study inception, the senior author had been in clinical practice for 8 years with a focus on shoulder arthroplasty. The surgical dates spanned from January 2016 through March 2020 and then January 2021 through January 2022. The intervening period was not included given the potential confounding effects of the COVID-19 pandemic. Institutional Review Board approval was obtained for this study [BLINDED].

The inclusion criteria included any patient who underwent an elective aTSA for noninflammatory arthritis using either a stemless or SS humeral implant. Exclusion criteria included anatomic arthroplasty done for inflammatory arthritis or those cases with missing or incomplete records. Demographic data including patient age, sex, side of operation, history of prior surgical intervention on the ipsilateral shoulder, BMI, and age-adjusted Charlson Comorbidity Index (ACCI) were collected. The ACCI is a validated instrument for predicting mortality based on comorbid disease and is a modification of the original CCI by adjusting risk by age.^14^ Surgical data included American Society of Anesthesiologists (ASA) score, implant type (stemless versus SS), glenoid design (augmented versus nonaugmented), surgical time, use of a Hemovac drain, administration of tranexamic acid (TXA), hospital LOS, VAS pain score on postoperative day 1 and day of discharge, and need for transfusion (intraoperatively or postoperatively). Surgical time was defined as the time from skin incision to the start of incision closure. The decision to administer a blood transfusion was based solely on the presence of patient symptoms (tachycardia and tachypnea) in the setting of anemia and no absolute transfusion trigger was used.

### Surgical Technique

The surgical technique was performed in a consistent and standard fashion in every case with a freehand anatomic neck osteotomy for stemless implants and use of an intramedullary cutting guide for SS humeral implants. A standard deltopectoral incision with a subscapularis peel was used in every case with repair through bone tunnels in the lesser tuberosity. The surgery was conducted under either general anesthesia alone or general anesthesia combined with regional anesthesia. None of the humeral implants were cemented. During the study period, there were no changes in the surgical plan from a stemless implant to a SS implant based on metaphyseal bone quality. A single loading dose of intravenous TXA was administered at a dose of 10 mg/kg over 10 minutes before incision, except where contraindicated by anesthesia. A Hemovac drain was placed in most of the cases and was removed 24 hours postoperatively. Chemical DVT thromboprophylaxis was not used during this study apart from early ambulation.

The Simpliciti stemless implant was used for all the stemless cases (Wright Medical). The SS implant was the Ascend Flex with proximal porous coating in all cases (Wright Medical). Before May 2018, only noncemented SS humeral implants were used, and after May 2018, only the stemless implants were used. The glenoid implants were hybrid with a press-fit polyethylene central peg with three peripheral cemented pegs. (Aequalis Perform Cortiloc or Perform+, Wright Medical) The need for posterior augmentation was based on presurgical planning and intraoperative assessment of posterior bone loss.

### Total Intraoperative Volume Loss

The preoperative and postoperative hematocrit levels were recorded. Total body volume was calculated using the formula of Nadler et al.^15^ Intraoperative TBVL (ITBVL) was calculated using the total body volume multiplied by the change in preoperative and postoperative hematocrit levels and adding back any intraoperative blood transfusion volume administered, as described by Sehat et al^16^ and previously used in clinical studies.^17^ For the purpose of calculating ITBVL, the preoperative hematocrit level was compared with the hematocrit level obtained early on the morning of postoperative day 1.

### Statistical Analysis

Unadjusted associations of demographic, clinical, and surgical variables with surgical time and total volume of ITBVL were examined using Spearman correlation coefficients for continuous variables and Wilcoxon rank sum tests for categorical variables. Nonparametric analysis of covariance was used to examine effects of stemless aTSA/SS aTSA on surgical time and total volume of intraoperative blood loss adjusted for demographic, clinical, and surgical variables. The following benchmark values for correlation coefficients were used: A value between 0 and 0.19 describes poor agreement; 0.20 to 0.39, fair agreement; 0.40 to 0.59, moderate agreement; 0.60 to 0.79, good agreement; and 0.80 to 1.00, excellent agreement. *P* < 0.05 was considered statistically significant. All statistical analyses were conducted using SAS version 9.4 (SAS).

## Results

There were 130 primary anatomic total shoulder arthroplasties conducted during the study period, of which 83 (64%) were SS and 47 (36%) used a stemless implant. The median age of a patient undergoing a SS arthroplasty was 62 years (IQR 56,69) compared with 63 years (IQR 55,72) for the stemless cohort (*P* = 0.43). There was a significantly higher proportion of women in the SS group (74% versus 47%, *P* = 0.002), but the remainder of the cohort characteristics were similar. The demographics of the SS and stemless groups are given in Table 1. Regarding surgical characteristics, 14% of the stemless aTSAs and 34% of the SS cohort used an augmented polyethylene implant (*P* = 0.24). No significant difference in TXA use was observed between the stemless and SS groups (90% versus 96%, *P* = 0.27) (Table 2).

**Table 1:** Patient Baseline Demographic Data

Clinical Parameters	Stemless aTSA, Median (IQR) (n = 83)	Short-stem aTSA, Median (IQR) (n = 47)	*P* Value
Age, yr	62 (56-69)	63 (55-72)	0.43
Sex, n (%)			0.002
Male	44 (53%)	12 (26%)	
Female	39 (47%)	35 (74%)	
Height, m	1.7 (1.6-1.8)	1.6 (1.6-1.7)	0.16
Weight, kg	94.3 (78-103)	81.6 (71.7-107)	0.12
BMI, kg/m^2^	33.2 (28.7-36.7)	31.2 (27.0-37.2)	0.40
Surgical side			0.23
Right	48 (58%)	22 (47%)	
Left	35 (42%)	25 (53%)	
ACCI	3 (1-4)	3 (2-4)	0.54
Prior ipsilateral arthroscopy			0.98
Yes	14 (17%)	8 (17%)	
No	69 (83%)	39 (83%)	

ACCI = age-adjusted Charlson Comorbidity Index, aTSA = anatomic total shoulder arthroplasty, BMI = body mass index, IQR = interquartile range

**Table 2:** Patient Surgical and Hospitalization Data

Surgical Parameters	Stemless aTSA, Median (IQR) (n = 83)	Short-stem aTSA, Median (IQR) (n = 47)	*P* Value
Surgical time (minutes)	111 (96-130)	137 (113-169)	<0.00001
Use of augmented polyethylene			
Yes	14 (14%)	12 (34%)	0.24
No	69 (86%)	35 (66%)	
ASA			0.61
1-2	35 (42%)	22 (47%)	
≥3	48 (58%)	25 (53%)	
Hemovac use			0.00007
Yes	51 (61%)	44 (94%)	
No	32 (39%)	3 (6%)	
Transexamic acid use			0.27
Yes	75 (90%)	45 (96%)	
No	8 (10%)	2 (4%)	
Anesthesia, n (%)			0.002
General alone	0 (0%)	6 (12%)	
General/regional	83 (100%)	41 (88%)	
Total intraoperative volume loss	298.3 (212.6-402.8)	359.7 (253.9-415.0)	0.05
Transfusion (No. of patients)			0.53
Yes	2	0	
No	81	47	
Length of stay	2 (1-2)	2 (2-3)	0.03
VAS pain score			
Postoperative day 1	2 (0-7)	6 (3-7)	0.0008
Day of discharge	0 (0-3)	4 (2-6)	<0.00001

aTSA = anatomic total shoulder arthroplasty, IQR = interquartile range

### Surgical Time

The median surgical time for the stemless group was 111 minutes (IQR 96,130) versus 137 minutes (IQR 113,169) for the SS group after univariate analysis (*P* < 0.00001) (Table 2). After multivariable analysis, female sex (109.5 versus 124 minutes, *P* < 0.001) and the use of a stemless implant (111 versus 137 minutes, *P* < 0.001) were found to be independently associated with shorter surgical time (Table 3). Furthermore, the ACCI was found to have a weak negative correlation with surgical time (r = −0.201, *P* = 0.009) (Table 3). A history of ipsilateral shoulder arthroscopy, ASA score, and BMI were not found to be independently associated with surgical time.

**Table 3:** Factors Associated With Surgical Time

	Surgical Time (min)	Unadjusted *P* Value	Adjusted *P* Value^a^
Implant type, median (IQR)		<0.001	<0.001
Stemless aTSA	111 (96-130)		
Short-stem aTSA	137 (113-169)		
Age (Spearman correlation)	r = −0.124	0.159	—^b^
Sex, median (IQR)		0.003	<0.001
Female	109.5 (93-137)		
Male	124 (111.5-145.5)		
BMI (Spearman correlation)	r = −0.084	0.340	—^b^
ASA, median (IQR)		0.068	—^b^
1-2	112.5 (97-133)		
≥3	123.5 (99.5-152.5)		
ACCI (Spearman correlation)	r = −0.201	0.022	0.009
Prior ipsilateral arthroscopy, median (IQR)		0.943	—^b^
No	117 (99-138)		
Yes	118.5 (92-158)		
Transexamic acid use, median (IQR)		0.238	—^b^
No	108.5 (92-139)		
Yes	118.5 (98.5-139.5)		
Anesthesia type, median (IQR)		0.495	—^b^
General alone	135.5 (98-138)		
General/regional	117 (98-139.5)		
Use of augmented polyethylene, median (IQR)		0.747	—^b^
No	117 (97.5-139)		
Yes	118 (104-140)		
Surgical side, median (IQR)		0.083	—^b^
Right	115 (97-133)		
Left	127 (98.5-149.5)		

ACCI = age-adjusted Charlson Comorbidity Index, ASA = American Society of Anesthesiologists, aTSA = anatomic total shoulder arthroplasty, IQR = interquartile range

a Adjusted *P* values were obtained from nonparametric analysis of covariance. This analysis was based on the ranks of surgical time.

b Variables did not remain in the nonparametric analysis of covariance model at a 0.05 significance level.

### Total Intraoperative Blood Loss

The median ITBVL for the stemless cohort was 298.3 mL (IQR 212.6, 402.8) versus 359.7 mL (IQR 253.9, 415.0) after univariate analysis (*P* = 0.052). After multivariable analysis, the difference remained significant (*P* = 0.005) (Table 4). Male sex and BMI were independently associated with increased TBVL, although the relationship with BMI was considered weak. TXA was used in 92.3% of all patients and was not found to be associated with the amount of ITBVL (*P* = 0.7). Increased surgical time, however, was found to be positively associated with ITBVL (r = 0.340, *P* < 0.0001). There were only two transfusions administered during the study period, both of which occurred in the stemless implant cohort. One transfusion was given intraoperatively and one postoperatively.

**Table 4:** Factors Associated With Total Volume of Intraoperative Blood Loss

	Total volume of intraoperative blood loss	Unadjusted *P* value	Adjusted *P* value^a^
Implant type, median (IQR)		0.052	0.005
Stemless aTSA	298.3 (212.6-402.8)		
Short-stem aTSA	359.7 (253.9-415.0)		
Age, Spearman correlation	r = −0.060	0.503	—^b^
Sex, median (IQR)		0.031	0.001
Female	299.7 (212.6-383.2)		
Male	357.2 (257.9-455.6)		
BMI (Spearman correlation)	r = 0.089	0.312	0.041
ASA, median (IQR)		0.806	—^b^
1-2	326.3 (229.2-412.1)		
≥3	323.0 (235.1-406.7)		
ACCI (Spearman correlation)	r = 0.022	0.803	—^b^
Prior ipsilateral arthroscopy (median (IQR))		0.631	—^b^
No	318.7 (218.1-412.1)		
Yes	346.2 (266.0-402.2)		
Hemovac use, median (IQR)		0.074	—^b^
No	274.8 (182.9-400.4)		
Yes	337.1 (237.8-412.1)		
Transexamic acid use, median (IQR)		0.704	—^b^
No	299.6 (280.7-400.4)		
Yes	326.3 (225.5-412.0)		
Anesthesia, median (IQR)		0.441	—^b^
General alone	246.9 (151.5-412.1)		
General/regional	326.3 (232.7-409.7)		
Use of augmented polyethylene, median (IQR)		0.344	—^b^
No	312.9 (232.7-404.9)		
Yes	349.7 (221.8-460.6)		
Surgical side, median (IQR)		0.482	—^b^
Right	318.1 (239.5-419.4)		
Left	325.4 (191.4-405.2)		

ACCI = age-adjusted Charlson Comorbidity Index, ASA = American Society of Anesthesiologists, IQR = interquartile range

a Adjusted *P* values were obtained from nonparametric analysis of covariance. This analysis was based on the ranks of total volume of intraoperative blood loss.

b Variables did not remain in the nonparametric analysis of covariance model at a 0.05 significance level.

### Length of Stay and Postoperative Pain

Patients who underwent stemless arthroplasty have a lower median hospital LOS compared with the SS cohort (2 days [IQR 1,2] versus 2 days [IQR 2,3], *P* = 0.03). Furthermore, stemless implants were associated with significantly lower VAS pain scores on both POD 1 (2 [IQR 0,7] versus 6 [IQR 3,7], *P* = 0.0008) and on the day of discharge (0 [IQR 0,3] versus 4 [IQR 2,6], *P* < 0.00001). Surgical time (r = 0.011, *P* = 0.901) and ITBVL (r = 0.008, *P* = 0.931) were not associated with hospital LOS.

## Discussion

The main findings of this study were that the use of stemless aTSA was associated with markedly shorter median surgical time and lower intraoperative blood volume loss when compared with the use of noncemented SS humeral implants. Male sex was similarly related to increased surgical time and blood loss. Furthermore, patients undergoing stemless aTSA had a shorter median hospital LOS and lower postoperative pain scores.

We found that the use of stemless aTSA was associated with markedly shorter surgical time compared with SS aTSA, with a median difference of 26 minutes. Wilson et al^9^ found that each 20-minute increase in surgical time increased the risk for all complications by 24%. Our findings are consistent with those of Anastasio et al^6^ who reported a nearly 14-minute shorter mean surgical time among the stemless cohort compared with the stemmed cohort, even when adjusting for BMI. It is not clear from their study, however, whether the stemmed implants were of the SL or SS design or whether any of the stemmed implants required the use of cement, factors which could influence the surgical time. Our results were also similar to those of Berth et al^4^ who reported a 14.7-minute shorter mean surgical time for their stemless cohort compared with patients undergoing cemented SL humeral aTSA. The lower surgical time in this study, even after adjusting for multiple patient and surgical demographics including sex, BMI, and use of an augmented polyethylene, was most likely related to the decreased preparation needed of the humeral canal. The surgical technique remained consistent throughout the study period, reducing the risk for confounders. Our findings were discordant with those of Wiater et al^18^ who, in a multicenter randomized controlled trial, reported no difference in surgical time between stemless and noncemented SS aTSAs. The implant manufacturer for that study differed from that in this study and underscores the fact that differences in surgical time may be specific to the device being used.

In this study, male sex, but not increased BMI, was found to be independently associated with increased surgical time. The increased surgical time among male patients has been surmised to result from the more extensive dissection required for the surgical exposure given the increased soft-tissue and muscle mass. A previous study similarly reported on markedly higher prolonged surgical time, defined as ≥150 minutes, in male patients and those with increasing BMI undergoing aTSA.^8^ In their national database study, however, the authors were unable to elucidate between aTSA and rTSA, the use of SL/SS/stemless implants, the specific implant manufacturer used, or whether cement was used during the implantation of the humeral implant, all factors which may have led to a difference from our results regarding the effect of BMI on surgical time. Furthermore, several previous studies have found that obesity was not markedly associated with surgical time after aTSA,^19,20^ although others have demonstrated the existence of this relationship among patients undergoing rTSA.^21^ Age-adjusted CCI was found to have an inverse relationship with surgical time, although it was only fair agreement. We surmise that there was an impetus to minimize surgical time for older patients with more advanced medical comorbidities undergoing aTSA, particularly in light of the potential for adverse effects under prolonged anesthesia.^22^ Horneff et al,^23^ in contrast, found that each additional point on the CCI was independently associated with a nearly 4-minute increased surgical time after aTSA. Finally, TXA use did not independently affect surgical time as demonstrated by others.^24^ Additional analysis of the importance of the effect of TXA on improving surgical visualization through reduced blood loss, and subsequently in lower surgical time, is warranted.

We found that intraoperative TBVL was markedly lower in the stemless aTSA cohort when compared with the SS patients, with an approximately 61-mL median difference. A recent systematic review demonstrated a nearly 100-mL lower blood loss after stemless aTSA compared with traditional SL stemmed implants.^13^ Berth et al^4^ similarly demonstrated a 97-mL lower mean blood loss in the stemless group. The use of the stemless humeral implant avoids violating the diaphysis of the humerus compared with SL and SS implants, likely contributing to the lower calculated blood loss. Our results differ from those of Malcherczyk et al^3^ who found no difference in total blood loss based on implant between a cohort of patients undergoing rTSA, stemmed aTSA, and stemless aTSA. Given the small number of stemless cases included, their study may have been underpowered to demonstrate a difference. It is unclear from our results whether the difference in intraoperative blood loss translates clinically into the need for more blood transfusions. Only a single intraoperative blood transfusion and one postoperative blood transfusion were administered to two individual patients in this study, both in the stemless group, for an overall prevalence of 1.5%. Our results are consistent with those of Berth et al^4^ who reported that no transfusions were administered in either the stemless or stemmed implant groups. By contrast, Malcherczyk et al^3^ reported a 14.4% transfusion rate among the rTSA and aTSA stemmed implant cohort and a zero% transfusion rate among the stemless cohort. The differences among the various studies are likely a result of the lack of an objective transfusion trigger at different institutions. In addition, the decision to administer a blood transfusion depends not just on isolated hematocrit levels but also on patient symptoms and comorbidities. Although not specifically assessed in this study, an alternative measure for determining the clinical importance of blood loss differences might be the calculation of the maximum allowable blood loss.^17^

Both BMI and male sex were found in this study to be associated with intraoperative TBVL. The need for more extensive soft-tissue dissection among patients with markedly elevated BMI^25^ and the presence of more lean muscle mass among male patients increasing surgical time^26,27^ both may contribute to increased blood loss. Perhaps expectedly, we found a fair positive correlation between increased surgical time and blood loss. Surgical time, BMI, and male sex have previously been reported as independent risk factors for perioperative blood loss.^3^ Interestingly, we did not find that the use of a dose of TXA at the onset of surgery was markedly associated with median intraoperative TBVL. Multiple previous studies have demonstrated the benefit of using TXA in reducing blood loss and the need for transfusion in total shoulder arthroplasty.^28,29^ It is likely that this study was underpowered to detect a clinical benefit of TXA because over 92% of our patients were given TXA, in addition to the different dosage used compared with previous studies. We did not find that the ASA classification was predictive of blood loss, a conclusion demonstrated by others in the setting of shoulder arthroplasty.^3^ Finally, the ACCI was not found to be associated with intraoperative TBVL in this study. The relationship between CCI and blood loss remains controversial in orthopaedic surgery, with one study demonstrating no correlation between CCI and intraoperative blood loss and another study demonstrating increased blood loss in total hip and knee patients with a CCI of > 3.^30,31^

There are certainly limitations to this study. All the procedures were conducted by a single surgeon, which may limit the generalizability of the reported findings. However, this methodology may potentially reduce the confounding effect of varying surgical techniques and experience. Furthermore, the estimation of surgical blood loss has been described using multiple published formulas relying on either hemoglobin or hematocrit levels. We have used a commonly described method in the orthopaedic literature, and calculating blood loss has generally been demonstrated to be more accurate than intraoperative direct measurements. We did not take into account the hidden blood volume loss into the surrounding soft tissues of the surgical site, which has previously been described by Sehat et al^16^ for lower extremity arthroplasty, nor did we report on TBVL. Finally, all the implants in this study were manufactured by the same company, and it is certainly possible that the results would be different if we used other implants that called for different surgical approaches or techniques. There was a difference in sex between the stemless and SS cohorts, although multivariable analysis was used to ascertain the independent association between implant design and both surgical time and blood loss, irrespective of patient sex. A strength of this study is the consistent surgical technique and postoperative protocols used. This study adds to the existing literature by comparing stemless implants with noncemented SS implants, as opposed to SL humeral implants, which have previously been reported in the literature.

## Conclusion

The use of stemless humeral implants was found in this single institutional retrospective study to be associated with markedly shorter surgical time and lower intraoperative TBVL compared with SS implants. Furthermore, the patients undergoing stemless aTSA had markedly shorter median hospital LOS and lower VAS pain scores on POD 1 and on discharge. In light of the existing literature demonstrating increased perioperative complications with increasing surgical time, our findings will be useful for future cost-based analysis analyzing the benefits of stemless aTSA.
